# Latest Trends in Outcome Measures in Dementia and Mild Cognitive Impairment Trials

**DOI:** 10.3390/brainsci12070922

**Published:** 2022-07-14

**Authors:** Divyani Garg, Anu Gupta, Ayush Agarwal, Biswamohan Mishra, Madakasira Vasantha Padma Srivastava, Aneesh Basheer, Venugopalan Y. Vishnu

**Affiliations:** 1 Department of Neurology, Vardhman Mahavir Medical College and Safdarjung Hospital, New Delhi 110029, India; divyanig@gmail.com; 2 Department of Neurology, All India Institute of Medical Sciences, New Delhi 110029, India; doctoranugupta1@gmail.com (A.G.); ayushthetaurian@gmail.com (A.A.); biswamohan26@gmail.com (B.M.); vasanthapadma123@gmail.com (M.V.P.S.); 3 Department of Medicine, DM Wayanad Institute of Medical Sciences (DM WIMS), Wayanad 673577, India; basheeraneesh@gmail.com

**Keywords:** outcome measures, dementia, mild cognitive impairment, MMSE, ADAS-Cog

## Abstract

Disease modification trials in dementia and mild cognitive impairment (MCI) have not met with success. One potential criticism of these trials is the lack of sensitive outcome measures. A large number of outcome measures have been employed in dementia and MCI trials. This review aims to describe and analyze the utility of cognitive/clinical outcome measures in Alzheimer’s disease (AD) and MCI trials. **Methods:** A PubMed search was conducted using relevant MeSH terms and exploded keywords. The search was confined to English language publications of human studies from the last five years which describe the latest trends in the use of outcome measures. **Results:** Despite broad use, the outcome measures employed are heterogeneous, with little data on correlations between scales. Another problem is that most studies are over-reliant on clinician/researcher assessment and cognitive outcomes, and there is a definite lack of stakeholder input. Finetuning of the paradigm is also required for people with early-stage disease, mild to moderate disease, and advanced dementia, as the outcome measures in these subgroups have varying relevance. Disease modification/prevention is an appropriate goal in early disease, whereas palliation and freedom from discomfort are paramount in later stages. The outcome measures selected must be suitable for and sensitive to these particular care goals. Although there is a shift to enrich MCI cohorts using a biomarker-based approach, the clinical relevance of such outcome measures remains uncertain. **Conclusions:** Outcome measures in dementia/MCI trials remain inhomogeneous and diverse, despite extensive use. Outcome measures fall within several paradigms, including cognitive, functional, quality-of-life, biomarker-based, and patient-reported outcome measures. The success of future disease-modifying trials is reliant to a large extent on the selection of outcome measures which combine all outcomes of clinical relevance as well as clinical meaning. Outcome measures should be tied to the type and stage of dementia and to the specific interventions employed.

## 1. Introduction

Around 47 million people live with dementia globally, with this number estimated to rise to 82 million by 2030 and 152 million by 2050 [1]. A number of common comorbidities are known to impact cognitive abilities, including diabetes, prediabetes [2,3] and hypertension [4]. Although the typical clinical dementia syndrome is readily recognizable, certain uncommon features, either in isolation or combination, may also herald the diagnosis. These include behavioural and psychological syndromes such as obsessive-compulsive disorder, repetitive questioning, minor accidents while driving, repeated falls, lack of personal attention, wandering, inappropriate use of objects, and rapid eye movement sleep behaviour disorder [5].

**Figure 1:**
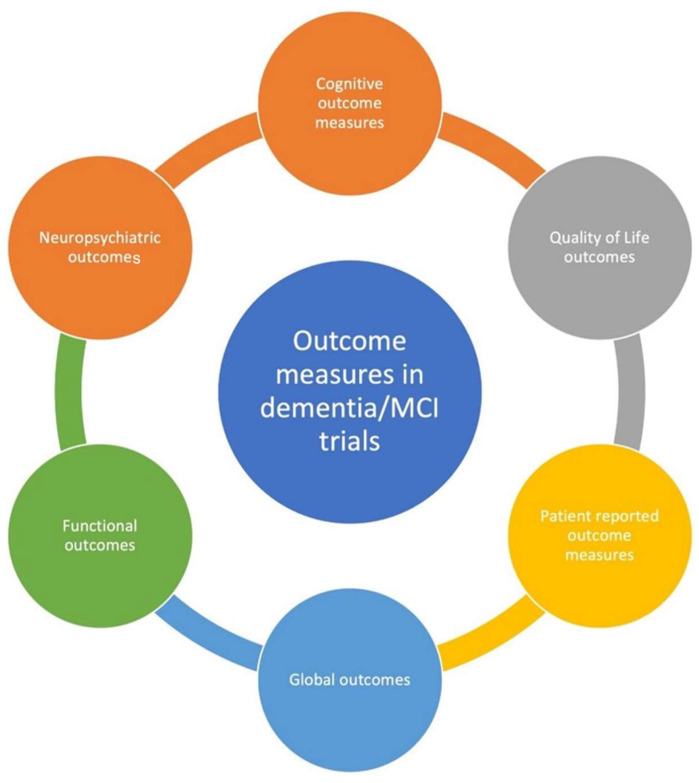
Types of outcome measures used in dementia and MCI clinical trials.

None of the disease modification therapy trials in Alzheimer’s disease (AD) or other forms of dementia have yielded success. One criticism of these trials has been the use of inappropriate outcome measures, including a perceived lack of stakeholder input and reliance on clinician/researcher assessments. Ref. [6] Outcome measures in dementia/MCI trials are numerous, largely heterogeneous, and are over-reliant on cognitive measures (Table 1). A review of 676 dementia and 129 MCI trial records found a large number of outcome measures, including several which were not standardised [7]. Multiple regulatory agencies have emphasized using cognitive, functional and global outcomes measures in AD, the dementia subtype most widely studied [8]. Selecting appropriate outcome measures in clinical trials depends on the study design and is crucial for ensuring the strength of the study. In dementia and mild cognitive impairment (MCI), both clinical and non-clinical outcomes have been employed (Figure 1). Non-clinical outcomes are based on radiological or other biomarker-based assessments. The reliability of the latter as surrogate outcome measures is debatable, as concordant results have not been achieved with clinical measures. Dementia outcomes focus on measuring either disease incidence and severity or the disease’s functional impact, which may be physical, cognitive, behavioural or psychological. Global assessment and quality of life-based scales may be appropriate, as dementia has a complex effect on the patient’s lived experience. Outcome measures that assess the impact on the caregiver/s are also increasingly encouraged. Ref. [9] In a similar vein, patient-reported outcome measures (PROMs) are also increasingly recommended, as they represent the patients’ perspectives and provide them an opportunity to share their concerns and viewpoints. However, PROMs continue to remain underutilized in dementia registries [10]. 

In this review, we aim to detail and analyze clinical and cognitive outcome measures that have been used in AD and MCI trials in order to document their strengths and weaknesses and utility as outcome measures in AD/MCI trials. We will not focus on biomarkers as outcome measures, since these require in-depth evaluation, which is beyond the scope of this review.

## 2. Search Methodology

We performed a search in the PubMed database on 30 April 2022 using the following MeSH terms: “dementia”, “cognitive impairment”, “cognitive dysfunction”, “outcome assessment, healthcare”, “patient reported outcome measures”, “Alzheimer disease”, and the keywords: dementia, mild cognitive impairment, minor neurocognitive disorder, outcome measure. The search was confined to studies on human subjects published in English and limited to the last five years. We included studies which reported the utility of outcome measures in dementia and MCI trials. The studies were screened based on title and abstract by two reviewers (DG, AG) and any conflict was resolved in concert with a third reviewer (VVY). After initial screening, the full text of eligible studies were accessed and assessed for their suitability. Relevant cross-references were also screened and assessed for eligibility. The initial search retrieved 1394 results. After screening the titles and abstracts, the full texts of 179 studies were accessed. A total of 53 studies were included in the final review.

## 3. Outcome Measures

### 3.1. Cognitive Outcome Measures

The majority of data on outcome measures in dementia emanates from studies on AD. Ref. [11] Less data is available for vascular dementia (VaD) and Dementia with Lewy Bodies (DLB). More than 300 cognitive measures have been utilized in AD/MCI studies. Some of the frequently used measures are described below. The psychometric properties of cognitive outcome measures are described in detail in a comprehensive review by Bossers et al. [11].

#### 3.1.1. Global Cognitive Function

*Mini-Mental State Examination (MMSE)*

The Mini-Mental State Examination (MMSE) is among the most frequently used cognitive outcome measure in dementia/MCI trials [7,12]. It has good internal consistency (Cronbach’s alpha > 0.7). Its test-retest reliability (intraclass correlations ICC 0.95), construct and criterion validity are also established [13,14].

For conversion from MCI to dementia, the sensitivity of baseline MMSE scores ranges from 23 to 76% and specificity from 40 to 94% [15]. For conversion from MCI to AD specifically, the sensitivity ranges from 27 to 89% and specificity from 32 to 90%. Floor and ceiling effects with preclinical AD and MCI are known to occur with the MMSE. MMSE may not be able to differentiate MCI from healthy controls [16]. Stand-alone MMSE may not be sufficient to predict MCI progression and may not be an optimal outcome in predementia and MCI trials. Although MMSE is a popular outcome measure, it was not designed as such, and data on its psychometric properties is limited [17].

Link: https://www.sciencedirect.com/science/article/abs/pii/0022395675900266?via%3Dihub (accessed on 11 July 2022).

*Alzheimer’s Disease Assessment Scale-Cognitive subscale (ADAS-COG)*

The ADAS was designed as a rating scale to assess the extent of both cognitive and non-cognitive issues in patients with AD [18]. Similarly to MMSE, it is a frequently used cognitive outcome measure in trials [7,12]. It consists of two subscales: ADAS-Cog and ADAS-Noncog. The cognitive subscale, ADAS-Cog, comprises 11 parts which are both patient-completed and assessor-based and test episodic memory, language, orientation and praxis. ADAS-Cog is one of the most widely used outcome measures in mild to moderate AD trials and has established utility in trials on cholinesterase inhibitors in dementia.

Severe floor and ceiling effects have been demonstrated with ADAS-Cog in MCI, mild and early AD, and preclinical states, with some of the item categories not yielding the intended results according to a Rasch analysis [19]. For these reasons, it has been suggested that ADAS-Cog is not an appropriate measure in pre-dementia or early dementia trials [18].

Link: https://www.fda.gov/media/122843/download (accessed on 11 July 2022).

*Montreal Cognitive Assessment (MoCA)*

MoCA is a 30-point screening tool and takes around 10 min to administer. Scores range between 0 and 30, and scores above 26 are abnormal. It has a sensitivity of 90% for detection of MCI (compared with 18% using MMSE) and 100% for mild AD. It also has excellent sensitivity (87%) [20]. MoCA also extends the cognitive evaluation offered by MMSE by including working memory, orientation, immediate and delayed memory, executive function and visuospatial abilities.

Link: https://www.mocatest.org/ (accessed on 11 July 2022).

Both MMSE and MOCA have been monetized, but there are special considerations for research studies and data collection.

*Saint Louis University Mental State (SLUMS)*

SLUMS is a tool for the assessment of mild cognitive impairment and dementia [21]. SLUMS was designed to overcome some of the limitations of MMSE [22]. It assesses memory, orientation and attention. In addition, executive function is assessed using the clock-drawing test and the animal recall task.

Link: https://www.slu.edu/medicine/internal-medicine/geriatric-medicine/aging-successfully/assessment-tools/mental-status-exam.php (accessed on 11 July 2022).

#### 3.1.2. Domain-Specific Cognitive Tests

These tests supplement the MMSE or the ADAS-Cog and cover specific cognitive subdomains. Some of the tests included in this category are the Trail Making Tests A and B, the Clock Drawing Test, the digit Span forward test, the digit Span backward test, word recognition tests, fluency tests, the Mohs Number Cancellation Test, the Rey Memory Test, the Digit Symbol Substitution Test, and the Buschke Selective Reminding test.

### 3.2. Functional Outcome Measures

Functional measures are underutilized in studies on MCI, with an estimated 16% using any measure of functionality [7].

#### 3.2.1. Alzheimer Disease Cooperative Study Activities of Daily Living Scale (ADCS-ADL)

The ADCS-ADL assesses basic and instrumental ADLs among patients with AD [23]. It may be clinician-administered or completed by the caregiver. The responses are based on the preceding four-week period. For patients with MMSE 0–15, the Alzheimer Disease Cooperative Study ADL-sev scale was developed [24]. It has also been adapted for patients with MCI.

Link: https://pubmed.ncbi.nlm.nih.gov/9236950/ (accessed on 11 July 2022).

#### 3.2.2. Barthel Index (BI)

BI is widely used in the assessment of basic ADLs, as it is simple to administer and score. Although it is validated and used extensively [25], validation in dementia is supported by limited evidence [26]. It consists of 10 items, and the cumulative score is calculated with scores ranging from 0 to 20. Although BI has good internal consistency, unidimensionality was not found to be robust. In a recent evaluation of BI in persons with dementia, multiple issues were found to interfere with its performance, including misfit items, item bias, measurement gaps etc. [26]. The authors suggested that BI needs further refinement before use with dementia patients.

Link: https://pubmed.ncbi.nlm.nih.gov/14258950/ (accessed on 11 July 2022).

#### 3.2.3. Disability Assessment for Dementia (DAD)

DAD is a questionnaire comprising 46 items which is administered to the caregiver and assesses basic and instrumental ADLs. It has a high internal consistency (Cronbach’s alpha > 0.8), good test-retest (ICC = 0.96) and inter-rater reliability (ICC = 0.95) [27].

Link: https://research.aota.org/ajot/article-abstract/53/5/471/4350/Development-of-a-Functional-Measure-for-Persons?redirectedFrom=fulltext (accessed on 11 July 2022).

### 3.3. Quality of Life Outcome Measures

#### 3.3.1. Quality of Life in Alzheimer’s Disease (QOL-AD)

The QOL-AD scale is a 13-item scale, with scores ranging from 13 to 52. Higher scores are indicative of better quality of life. The scale has high content validity [28,29]. and the interrater reliability is also good, with Cohen’s kappa values > 0.70. Likewise, it has good internal consistency, with a Cronbach’s alpha coefficient of 0.82.

Link: https://journals.lww.com/psychosomaticmedicine/Abstract/2002/05000/Assessing_Quality_of_Life_in_Older_Adults_With.16.aspx (accessed on 11 July 2022).

#### 3.3.2. Quality of Life in Late-Stage Dementia (QUALID)

QUALID is an 11-item scale, with total scores in the range 11–55. It was created from a subset of a larger number of items by Albert et al. [30]. Lower scores are consistent with a better quality of life [31]. The items are rated on a five-point Likert scale. It can be used for persons with an MMSE score ≥ 3 and was initially designed for use in patients with severe dementia in long-term care facilities. It is an assessor-administered questionnaire, which requires contact with the patient for at least 30 h. It is administered over an observation period of one week. It is modulated by the effect of antidepressant or antipsychotic drugs, leading to lower scores.

QUALID has been found to have good internal consistency, with a Cronbach’s alpha of 0.77. It has good test-retest and interrater reliability (ICC = 0.8).

Link: https://pubmed.ncbi.nlm.nih.gov/12818023/ (accessed on 11 July 2022).

#### 3.3.3. Quality of Life for People with Dementia (QUALIDEM)

QUALIDEM is a validated, questionnaire-based tool for assessing the quality of life of patients in residential care [32]. It is administered by professional caregivers.

Link: https://onlinelibrary.wiley.com/doi/10.1002/gps.1713 (accessed on 11 July 2022).

### 3.4. Neuropsychiatric Outcome Measures

#### 3.4.1. Neuropsychiatric Inventory (NPI)

The NPI is a popular scale that assesses 12 behavioural and psychiatric issues that occur in persons with dementia [33]. It has been established as both valid and reliable [34]. It has been translated into more than 40 languages, enabling wide usage. Four major versions have been developed [34]. The original 10-item scale was expanded to include sleep and appetite changes, to form the 12-item NPI. Another version, NPI-12 with integrated caregiver distress, is used most frequently. The NPI, Nursing Home version (NPI-NH), is a modification for use in residential facilities. It is suitable for use in settings where non-family caregivers are the informants.

Link: https://download.lww.com/wolterskluwer_vitalstream_com/permalink/cont/a/cont_21_3_2015_02_26_kaufer_2015-10_sdc2.pdf (accessed on 11 July 2022).

#### 3.4.2. Brief Psychiatric Rating Scale (BPRS)

BPRS was developed for patients with schizophrenia and was extrapolated for use with patients with dementia. However, the delusions and hallucinations observed in schizophrenia differ from those prevalent in neurodegenerative disorders.

Link: https://www.smchealth.org/sites/main/files/file-attachments/bprsform.pdf?1497977629 (accessed on 11 July 2022).

#### 3.4.3. Hamilton Rating Scale for Depression

Certain aspects of the scale, such as weight and appetite changes and social withdrawal, may occur in persons with dementia in the absence of depression.

Several other scales used in clinical trials include the Cohen-Mansfield Agitation Inventory, the Geriatric Depression Scale and the Cornell Scale for Depression in Dementia.

Link: https://jnnp.bmj.com/content/23/1/56.long (accessed on 11 July 2022).

### 3.5. Global Outcomes Measures

#### 3.5.1. Clinician’s Interview-Based Impression of Change Plus Caregiver Interview (CIBIC-Plus)

CIBIC-Plus is a global outcome measure widely used in trials among patients with advanced dementia as a co-primary outcome [35,36,37,38]. It is based on a seven-point rating scale based on clinician judgement and a semi-structured interview to assess cognition, behaviour and function. There has been a decline in its use with a shift towards using more objective measures. In a recent trial, CIBIC-Plus compared favorably to goal attainment scaling (GAS) for clinical meaningfulness [39].

Link: https://pubmed.ncbi.nlm.nih.gov/9236949/ (accessed on 11 July 2022).

#### 3.5.2. Clinical Dementia Rating (CDR)

CDR is a global assessment tool which provides global and Sum of Boxes (SOB) scores [36,40]. Global scores are used for the assessment of dementia severity. However, it contains no measure of behavioural issues.

Link: https://www.cambridge.org/core/journals/the-british-journal-of-psychiatry/article/abs/new-clinical-scale-for-the-staging-of-dementia/D1AAE7A0836C1E36B450461613521D20 (accessed on 11 July 2022).

#### 3.5.3. Clinical Dementia Rating-Sum of Boxes (CDR-SOB)

CDR-SOB is derived from the CDR. CDR-SOB has been assessed as a single primary outcome measure in mild to moderate AD. It showed good internal consistency (Cronbach’s alpha 0.88) and acceptable validity. It also has low variability, leading to smaller sample sizes compared with ADAS-Cog [41]. CDR-SOB is easier to calculate (Global CDR calculation is demanding and subject to errors unless one uses the online calculators) and better at detecting change across stages of dementia.

Link: https://pubmed.ncbi.nlm.nih.gov/3389756/ (accessed on 11 July 2022).

#### 3.5.4. Clinical Global Impressions (CGI)

The CGI scales measure the severity of symptoms (CGI-S) or changes in several psychiatric conditions (CGI-C) [42].

### 3.6. Patient-Reported Outcome Measures

Growing significance is attached to patient-reported health-related quality of life (HRQoL) measures in chronic disorders. PROMs are a tool to assess HRQoL as reported by the patients. Ayton et al. identified seven dementia-specific PROMs in a scoping review [10]. These included: Alzheimer’s Disease-Related Quality of Life (ADRQOL), Bath Assessment of Subjective Quality of Life in Dementia (BASQID), Dementia Quality of Life measures (D-QoL), Quality of Life in Alzheimer’s Disease (QoL-AD), QUALID and QUALIDEM. Of these, QUALID has been used in advanced dementia. ADRQOL and QUALIDEM may be caregiver-administered so may also be useful in late-stage dementia.

## 4. Early-Stage AD

“Prodromal AD” refers to both MCI and pre-MCI, which is defined as a state of cognitive dysfunction which does not meet the criteria for MCI. These individuals are at greater risk for the development of AD [43]. Another ‘preclinical’ group may be identified based on an increased risk for AD by genetic or biomarker-based assessment. These populations are targets for AD prevention by disease modification. Prodromal AD assessment requires newer sensitive measures rather than traditional neuropsychological tests. The outcome measures in these groups should possess “bi-directional sensitivity, longitudinal tracking, and sensitivity to impairment” [44]. The widely used ADAS-Cog is not suited to the MCI population for several reasons discussed above. Tests of metacognition, social cognition, and prospective memory are steps in this direction [44]. A few examples include the Loewenstein-Acevedo Scales of Semantic Interference and Learning (LASSI-L) [45], and short-term visual memory binding (SVMB) [46].

Despite the well-known importance of quality of life, caregiver burden and other functional outcomes in MCI and early dementia trials, cognitive outcomes continue to be widely used [47], the reason being that detection of functional impairment in early stages also requires instruments sensitive to very subtle functional changes. Tests for financial capacity, performance-based skill assessments and computerized assessments based on virtual reality and video technology are emerging in this area [44]. However, till the time we have a single holistic outcome measure for MCI, the creation and validation of cognitive composite scores (e.g., a composite score including delayed word list recall, logical memory, category fluency, tests of processing speed, tests of performance IQ etc.) may be the best option [48].

## 5. Mild to Moderate AD

A systematic review identified 81 outcome measures used across trials on mild to moderate AD [12,49]. The most widely used were measures of cognition and global assessment. Others included ADLs, biological markers and neuropsychological outcomes. The authors recommended the use of either ADAS-Cog or MMSE for cognitive outcomes. Structural MRI was judged to be a core outcome. For neuropsychiatric symptoms, NPI was recommended. DEMQOL was recommended for the assessment of the quality of life. Fluid biomarkers and ADLs were not recommended in this consensus statement. Although the consensus panel did not recommend the global outcome as a core outcome, CDR was considered appropriate if it was to be used. Overall, both cognition and biomarkers were recommended as core outcomes in persons with mild to moderate AD.

## 6. Advanced AD

A different paradigm applies to the application of outcome measures in individuals with advanced dementia, with a shift to palliative care and ensuring the quality of life [50]. For assessing quality of life, QUALID may be appropriate, as it was designed for patients with advanced dementia in residential care. Comfort is another important target in this group. Discomfort Scale for Dementia of the Alzheimer’s type (DS-DAT), which is based on interviews of nurses caring for persons with dementia, has nine items [51]. It has good psychometric properties, with good internal consistency and interrater reliability. Similarly, other targets in this population include assessment of engagement, pain, behaviour, agitation, apathy, rejection of care, and respiratory issues. Scales are also available to assess end-of-life care outcomes in advanced dementia, such as the Mini-Suffering State Examination (MSSE) [52] and Comfort Assessment in Dying with Dementia (CAD-EOLD) [53].

## 7. Future Directions and Perspectives

There is wide heterogeneity in outcome measures used in dementia research. High variability in outcome measures usually results in a large sample size. Although multiple efforts have been made to achieve consensus on measurement scales and outcome instruments, these efforts have not produced consistent conclusions. MMSE still remains the cornerstone of outcome measures in dementia/MCI trials, despite not being designed as an outcome measure and lacking well described psychometric properties [7]. There have also been endeavors to understand how scales relate to each other and describe the correlation so that clinicians may be able to compare different scales [54], but such data remains limited. There is an urgent need for homogeneous and standardized measures in this field. It should also be emphasized that outcome measures likely need to be tailored to the type of dementia, and a one-size-fits-all approach may not be applicable, considering that different forms of dementia have varying impacts on patients.

From the Indian perspective, the ICMR-Neurocognitive toolbox (ICMR-NCTB) has been designed to diagnose dementia and MCI and can be applied across a wide linguistic and educational range in India. It has been validated for the diagnosis of MCI in India and has a sensitivity of 81.1% and specificity of 88.8% [55]. It has also been validated for the diagnosis of dementia in India, with a sensitivity and specificity of 70–100% [56] in five Indian languages (Hindi, Bengali, Telugu, Kannada, Malayalam). The ICMR-NCTB comprises multiple tests to evaluate multiple cognitive domains, including attention-executive function, memory, language, and visuospatial function, along with questionnaires for depression, functional activities and quality of life. Future studies should assess the utility of this tool as an outcome measure across the spectrum of MCI and dementia.

There is also growing interest in the clinical meaningfulness of outcome measures based on what persons living with dementia and their caregivers uphold as meaningful to their lived experience. A ‘Clinically meaningful’ effect is a large, statistically significant effect on the patient’s perception and living. Apart from memory and cognition, ADLs, mental and social health, quality of life, caregiver burden, and maintaining patient identity and independence are important to patients and caregivers [57,58], and outcome measures must include these perspectives. However, only 13% of dementia trials have included quality of life measures [7]. Hence, historically, dementia trials use the co-primary endpoint approach, that is, the use of at least two outcome measures for cognitive and functional or global impairment. However, this approach may not be valid for predementia trials due to a lack of significant functional impairment.

## 8. Conclusions

A large number of outcome measures have been described and evaluated in dementia and MCI trials, encompassing cognitive and non-cognitive outcomes. The large majority of current trials are still reliant on global measures such as MMSE and ADAS-Cog. Although there is a shift to enrich MCI cohorts using a biomarker-based approach, the clinical meaningfulness of such outcome measures remains uncertain. The success of future disease-modifying trials is reliant to a large extent on the selection of outcome measures which combine all outcomes of clinical relevance as well as clinical meaning. Outcome measures should be tied to the type and stage of dementia and to the specific interventions employed.

## Figures and Tables

**Table 1:** Summary table of some outcome measures used in AD and MCI trials.

Outcome Measure	Description	Stage of Dementia	Benefits	Deficiencies
* Mini-Mental State Examination (MMSE) *	Most commonly used cognitive measure in trials; scored on 30 points	Mild to moderate	Easy to administer, rapid	Floor and ceiling effects; Stand-alone MMSE may not be sufficient to predict MCI progression; limited data on psychometric properties; affected by level of education
* Alzheimer’s Disease Assessment Scale-Cognitive subscale (ADAS-COG) *	More detailed than MMSE	Mild to moderate	Detailed evaluation, may be more sensitive to subtle changes	Tedious, does not sufficiently assess attention, planning, working memory, executive function
* Montreal Cognitive Assessment (MoCA) *	30-point screening tool requiring around 10 min	All stages	Extends the cognitive evaluation offered by MMSE by including working memory, orientation, immediate and delayed memory, executive function and visuospatial abilities	Requires more time to administer than MMSE
*Saint Louis University Mental State (SLUMS)*	11-item scale, scores < 27 indicate cognitive impairment	All stages	Assesses executive function	Requires more time to administer than MMSE
* Alzheimer Disease Cooperative Study Activities of Daily Living scale (ADCS-ADL) *	Assesses basic and instrumental ADLs	All stages	For patients with MMSE 0–15, the Alzheimer Disease Cooperative Study ADL-sev scale was developed; Also adapted for patients with MCI	
* Disability Assessment for Dementia (DAD) *	46 items via a questionnaire administered to the caregiver; Assesses basic and instrumental ADLs	All stages	Good intra- and interrater reliability	
* Quality of Life in Alzheimer’s disease (QOL-AD) *	13-item scale, with scores ranging from 13 to 52	Mild to moderate dementia	Excellent internal consistency and reliability	Cannot be applied for individuals with MMSE < 10
* Quality of life in late-stage dementia (QUALID) *	11-item scale, with total scores ranging from 11 to 55; scored over one week	All stages	Good internal consistency, inter-rater reliability, and test-retest reliability	Sensitive to effects of medications, such as neuroleptics
* Quality of Life for People with Dementia (QUALIDEM) *	Developed for patients in residential settings	All stages	21/40 items suitable for patients with very advanced dementia	
*Neuropsychiatric Inventory (NPI)*	Covers 12 neuropsychiatric symptoms over one month retrospectively	Mild to moderate dementia	Good validity and reliability	May be affected by recall bias
* Clinician’s Interview-Based Impression of Change plus caregiver interview (CIBIC-Plus) *	Seven-point rating scale which measures global functioning	All stages	CIBIC-Plus may compare favourably to goal attainment scaling (GAS) for clinical meaningfulness	May have subjective bias
* Clinical Dementia Rating (CDR) *	CDR is a global assessment tool with a five-point scale, which provides global and Sum of Boxes (SOB) scores in six cognitive domains	All stages	Recommended as a core outcome for global function	Does not contain measures of behavioral issues

## Data Availability

All data generated or analyzed during this study are included in this published article.
